# Shorter length of hospital stay for hip fracture in those with dementia and without a known diagnosis of osteoporosis in the USA

**DOI:** 10.1186/s12877-020-01924-x

**Published:** 2020-12-03

**Authors:** Rafia S. Rasu, Rana Zalmai, Aliza R. Karpes Matusevich, Suzanne L. Hunt, Milind A. Phadnis, Nahid Rianon

**Affiliations:** 1 Department of Pharmacotherapy,, University of North Texas Health Science Center, College of Pharmacy, Fort Worth, TX USA; 2 Department of Health Behavior and Health Systems, University of North Texas Health Science Center, School of Public Health, Fort Worth, TX USA; 3 Department of Biostatistics and Data Science, University of Kansas Medical Center, School of Medicine, Kansas City, Kansas USA; 4 Department of Family and Community Medicine, The University of Texas Health Science Center at Houston, McGovern Medical School, 6341 Fannin Street, #JJL 324C, Houston, TX 77030 USA; 5 Division of Geriatric and Palliative Medicine, Department of Internal Medicine, The University of Texas Health Science Center at Houston, McGovern Medical School, TX Houston, USA

**Keywords:** Hip fracture, Dementia, Length of stay, Osteoporosis, Costs, Medicare, Older adults

## Abstract

**Background:**

About 50% of all hospitalized fragility fracture cases in older Americans are hip fractures. Approximately 3/4 of fracture-related costs in the USA are attributable to hip fractures, and these are mostly covered by Medicare. Hip fracture patients with dementia, including Alzheimer’s disease, have worse health outcomes including longer hospital length of stay (LOS) and charges. LOS and hospital charges for dementia patients are usually higher than for those without dementia. Research describing LOS and acute care charges for hip fractures has mostly focused on these outcomes in trauma patients without a known pre-admission diagnosis of osteoporosis (OP). Lack of documented diagnosis put patients at risk of not having an appropriate treatment plan for OP. Whether having a diagnosis of OP would have an effect on hospital outcomes in dementia patients has not been explored. We aim to investigate whether having a diagnosis of OP, dementia, or both has an effect on LOS and hospital charges. In addition, we also report prevalence of common comorbidities in the study population and their effects on hospital outcomes.

**Methods:**

We conducted a cross-sectional analysis of claims data (2012–2013) for 2175 Medicare beneficiaries **(**≥65 years) in the USA.

**Results:**

Compared to those without OP or dementia, patients with demenia only had a shorter LOS (by 5%; *P* = .04). Median LOS was 6 days (interquartile range [IQR]: 5–7), and the median hospital charges were $45,100 (IQR: 31,500 − 65,600). In general, White patients had a shorter LOS (by 7%), and those with CHF and ischemic heart disease (IHD) had longer LOS (by 7 and 4%, respectively). Hospital charges were 6% lower for women, and 16% lower for White patients.

**Conclusion:**

This is the first study evaluating LOS in dementia in the context of hip fracture which also disagrees with previous reporting about longer LOS in dementia patients. Patients with CHF and IHD remains at high risk for longer LOS regardless of their diagnosis of dementia or OP.

## Background

Hospital related outcomes, including length of stay (LOS) and hospital charges, are significant challenges faced by the health care system as the incidence of hip fracture rises beyond what is predicted every year [1]. About 50% of all hospitalized fragility fracture cases in older Americans (≥65 years old) are hip fractures [2], which are responsible for more than 300,000 hospitalizations each year [3–5]. In the USA, approximately 3/4 of fracture-related costs are attributable to hip fractures, and these are mostly covered by Medicare [1]. The rates of hip fracture rise exponentially between the 6th and 8th decades of life [6], and the rate doubles in patients with dementia [7]. While about 50% of patients with hip fracture may potentially return to their pre-fracture functional level, patients with dementia suffer worse post-fracture functional impairment [8, 9]. Although limited, a few research studies reported the benefit of post-hip fracture rehabilitation in dementia patients [7, 10, 11]. Yet, these patients are often left out from rehabilitation, because they are not recognized as good candidates [7]. Discharge coordination in association with rehabilitation and destination sites are reported barriers for a longer hospital LOS [11]; dementia adds to this process by increasing LOS and hospital charges [12–15].

Presence of comorbidities measured by the Charlson Comorbidity Index (CCI) has been associated with higher risk of hip fracture and higher post-fracture mortality [5, 16]. While dementia, including Alzheimer’s diseases, is part of the CCI calculator, it is not clear how the presence of dementia may affect hospital outcomes in older patients with hip fractures. A fracture is often the first sign of osteoporosis (OP) and is thus diagnosed clinically during post-hip fracture hospitalization [17]. Research describing LOS and acute care charges for hip fractures has mostly focused on these outcomes in trauma patients without a known pre-admission diagnosis of OP. Yet, an appropriate disease specific follow up plan may be missed if a diagnosis is not documented in chart [18]. Whether having a pre-fracture diagnosis of OP would have an effect on hospital outcomes in dementia patients has not been explored.

LOS and hospital charges for dementia patients are usually higher than those without dementia [12–14]. One US study has mentioned shorter LOS in patients with or without dementia after TAVR [19]. However, there is a gap in knowledge about LOS in patients with dementia who suffered a hip fracture. Furthermore, hip fracture patients with dementia have a higher risk of discharge to nursing homes (NHs) and worse health outcomes [16]. Previous research reported fracture-related health care costs that included hospital charges and follow-up care for up to 6 months after the hip fracture [20]. However, hospital charges during acute care for patients with a hip fracture with or without dementia, and with or without a pre-fracture diagnosis of OP, has not been discussed.

In this study, we aim to investigate whether having a diagnosis of OP, dementia, or both has an effect on the following hospital outcomes: i) LOS, ii) hospital charges, and iii) discharge destinations in Medicare beneficiaries who suffered a hip fracture in the USA. Specifically, we report the outcomes for four patient groups: i) having a diagnosis of dementia, ii) having a diagnosis of OP, ii) having a diagnosis of both dementia and OP, and iv) having no diagnosis of dementia or OP. In addition, we report prevalence of common comorbidities in the study population and their effects on hospital outcomes.

## Methods

### Data source

Claims data for a random sample of 100,000 Medicare beneficiaries (2012–2013) from the Centers for Medicare and Medicaid Services (CMS) were used as the data source for the current study. Medicare data provide beneficiary summary files, claims, and assessment data that are linked by a unique beneficiary identification number across different files. We used several files to obtain information on comorbidities, eligibility, and inpatient admissions. Information on inpatient claims regarding hospitalization due to hip fracture was obtained using the inpatient base claims version j file. Comorbidities were determined using the Medicare Master Beneficiary Chronic Conditions file, which contains the first occurrence date of the chronic condition. If the first occurrence date of the chronic condition occurred prior to the hip fracture, we considered the comorbidity to be present. Medicare eligibility was determined using the Master Beneficiary Summary files for Parts A, B, and D.

### Study sample

All Medicare beneficiaries with a hip fracture between 7/1/2012 and 12/31/2013 were identified from CMS inpatient data. As part of a larger study evaluating post-fracture function, only those with a long-term minimum data set (MDS) assessment after hospital discharge were included in the sample. Furthermore, we only included beneficiaries who were eligible for Medicare Part A/B/D for at least 180 days prior to the hip fracture. Lastly, we excluded from our sample all patients under 65 years old.

### Outcome variables

Our primary outcomes variables were LOS and hospital charges for the hip fracture. LOS was the number of days spent in the hospital for hip fracture, including the admission and discharge dates. Hospital charges were provided as a total of all services included on the institutional claim for hip fracture (clm_tot_chrg_amt) (codes for hip fractures are listed in supplementary Table 1). Charges from physicians were not included. Our secondary outcome, discharge destination, was determined from the discharge status code (ptnt_dschrg_stud_cd) on the claim indicating the final disposition of the patient after discharge for the hip fracture event (supplementary Table 2). The 43 possible codes for discharge status were grouped into 6 categories: Home, Skilled Nursing Facility/Nursing Homes (SNF/NH), Inpatient Rehabilitation, Acute Care, Died/Hospice, and Other.

### Other variables

We collected demographic information from the Master Beneficiary Summary Part A/B file, including sex, race, and age, which was expressed in years and calculated as of the end of the calendar year when the hip fracture occurred. The seven codes for race were categorized as either White or Other, since a majority of the patients in this study was White. The Medicare Master Beneficiary Chronic Conditions file was used to identify the presence of Alzheimer’s disease (AD) or OP. Due to limitation of use of claims data, we used the term dementia (this includes dementia in general including AD) for the current manuscript. If the first occurrence date for dementia or OP was prior to the date of hip fracture, then we counted these patients in the dementia group or the OP group. Similarly, in this manner we determined other comorbidities such as congestive heart failure (CHF), chronic kidney disease (CKD), diabetes, hypertension, ischemic heart disease (IHD), and acute myocardial infarction (AMI).

### Data analysis plan

Initially, a univariate test of equality for LOS among the four clinical groups−OP only, dementia only, both diagnoses, and neither diagnosis−was planned. The LOS was skewed, thus the nonparametric Kruskal-Wallis test was used. Possible associations between group and race, sex, and comorbidities were explored using Chi-square tests with three degrees of freedom (df). The null hypothesis that the median age at the time of fracture was the same for all four groups was tested by a Kruskall-Wallis test. Beneficiary characteristics significantly associated with the clinical group were used as covariates in a multiple linear regression. Surgical repair was also included in the model as it directly impacts LOS. Due to the asymmetric distribution of LOS, the log transformation of LOS was modeled. A full model was chosen to avoid underfitting and the accompanying bias. To satisfy model assumptions, a generalized gamma regression model was used to model the natural log of LOS across the groups and control for sex, race, age at fracture, and comorbidities. The distribution of the residuals was specified as gamma.

We followed a similar process for the analysis of hospital charges. The distribution was skewed, and thus a Kruskal-Wallis test of equality was used to compare median hospital charges for the four groups. Next, a generalized linear regression was used to examine the log transformation of total hospital charges across groups in the presence of covariates. Surgical repair was included in the model given its relationship to cost. Model assumptions were met by using a generalized gamma regression model for the natural log of hospital charges and specifying a gamma distribution for the residuals.

The distribution of discharge status in the four groups was compared using a Chi-square (row mean scores differ) test with df = 3. The data were stratified by clinical group.

A statistical significance level of .05 was used for all tests, and no adjustments were made for multiple tests. The analysis for this project was generated using SAS software, Version 9.4 of the SAS System for Windows (SAS Institute, Inc., Cary, NC).

This study was exempt by the Institutional Review Board (IRB) according to national regulations by the IRB of the University of North Texas Health Science Center. University of Kansas Medical Center had a data use agreement with Center for Medicare and Medicaid Services (CMS) and also had IRB approval for CMS data analysis.

## Results

**Fig. 1:**
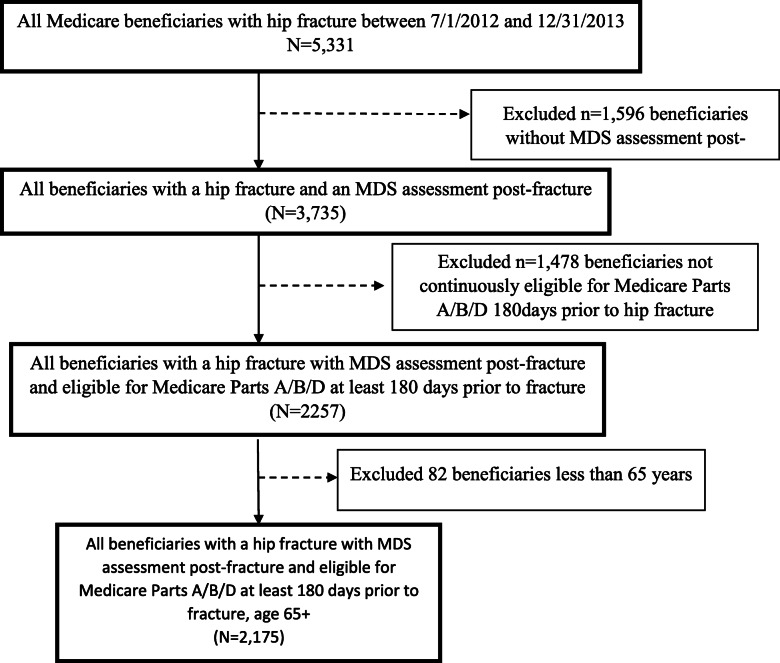
Flow chart describing sample selection. Abbreviations: MDS, minimum data set

The initial sample consisted of 5331 Medicare beneficiaries aged ≥65 years, admitted with a hip fracture between July 1, 2012, and December 31, 2013. A total of 2175 patients met all inclusion criteria and were included in our analysis (Fig. 1). Figure 1 is showing the flowchart of the study cohort pulled from CMS claims datasets of Medicare beneficiaries. Several analytical variables were created using real-world claims and coding to generate an accurate study cohort and report our study findings in detail. The supplementary tables include ICD-9-CM codes for fracture type and surgical repair and categorization of important analytical variables used for this study analyses.

The results presented in Table 1 describe demographic and clinical characteristics, including type of hip fractures and chronic comorbidities, in the study population, which mostly consisted of women (77%) and patients with a White race/ethnic background (92%). The group with no OP and no dementia (reference group for our analysis) was the largest (40%) in number and the youngest, with more than half of them (54%) in the younger (65–84 years) age group (Table 1). The different fracture types that occurred in these patients were femoral neck (51%), intertrochanteric (44%), and subtrochanteric (5%). Inter-trochanteric hip fracture was most common among the groups with OP alone or combined with dementia, whereas femoral neck was the most frequent site of fracture among those without any OP (dementia only and the reference group). Close to 90% (*n* = 1976) of patients underwent surgical treatment for their hip fractures, but there was a tendency to fewer surgeries in the dementia groups (88% versus 91–92%). Hypertension was the most common comorbidity amongst all, followed by CHF, which was diagnosed most often among those with OP alone or in combination with dementia.

**Table 1:** Population characteristics for the four clinical groups

Characteristic	All N (%)	Clinical groups				***P*** *
		Dementia Only N (%)	OP Only N (%)	Both OP and dementia N (%)	Neither OP nor dementia N (%)	
Sample size	2175 (100)	279 (13)	774 (36)	247 (11)	875 (40)	
Sex, Female	1679 (77)	176 (63)	708 (91)	224 (90)	571 (65)	<.01
Race, White	2006 (92)	240 (86)	731 (94)	227 (91)	808 (92)	<.01
**Age**						
65–84 years	1030 (47)	123 (44)	334 (43)	93 (37)	480 (54)	<.01
85+ years	1145 (53)	156 (56)	440 (56)	154 (62)	395 (45)	
Median (IQR)	85 (79, 90)	86 (80, 90)	86 (80, 90)	87 (83, 91)	83 (77, 89)	
**Fracture type**						<.01
Femoral Neck	1114 (51)	161 (57)	362 (46)	113 (45)	478 (54)	
Intertrochanteric	960 (44)	109 (39)	372 (48)	126 (51)	353 (40)	
Subtrochanteric	101 (5)	< 10 (3)	40 (5)	< 10 (3)	44 (5)	
Surgical repair of fracture	1980 (91)	247 (88)	710 (91)	218 (88)	805(92)	.12
**Comorbidities**						
Congestive heart failure	1097 (50)	137 (49)	404 (52)	148 (59)	408 (46)	<.01
Chronic kidney disease	848 (39)	110 (39)	291 (37)	113(45)	334 (38)	.13
Diabetes	931 (43)	120 (43)	308 (39)	105 (42)	398 (45)	.14
Hypertension	2021 (93)	262 (93)	729 (94)	236 (95)	794 (90)	.01
Acute myocardial infarction	205 (9)	19 (6)	66 (8)	23 (9)	97 (11)	.12
Ischemic Heart Disease	1474 (68)	193 (69)	530 (68)	183 (74)	568 (64)	.04
Hyperlipidemia	1767 (81)	232 (83)	656 (84)	198 (80)	681 (77)	<.01
Depression	1154 (53)	190 (68)	391 (50)	180 (72)	393 (44)	<.01
Arthritis^a^	1612 (74)	193 (69)	625 (80)	214 (86)	580 (66)	<.01

*Abbreviations*: *IQR* interquartile range, *OP* osteoporosi

^*^P values are for Chi-square tests examining the distribution across the four groups, except for age: Kruskal-Wallis was used

^a^Rheumatoid arthritis or osteoarthritis

Race, sex, CHF, depression, hypertension, hyperlipidemia, IHD, and rheumatoid arthritis/osteoarthritis demonstrated statistically significant differences in at least one of the four clinical groups. AMI, CKD, and diabetes were not distributed differently across the groups (Table 1).

The average age at the time of the hip fracture was 84 years for all patients; the average age within each group was as follows: 85 years for the dementia group, 85 years for the OP group, 87 years for the dementia/OP group, and 83 years for the neither dementia/OP group.

Table 2 reports the distribution of LOS, discharge destination, and hospital charges by the four clinical groups. LOS and hospital charges were similar for all groups (Table 2). Median LOS was 6 days (IQR: 5–7), and hospital discharge status was distributed similarly across each of the four groups (Cochran-Mantel-Haenszel = 6.8210; df = 3; *P* = 0.0778; row means differ). Median hospital charges were $45,100 (IQR: 31,500 − 65,600). Most beneficiaries were discharged to a nursing facility, including a SNF (*n* = 1777; 81.5%), while about 10.5% proceeded to inpatient rehabilitation (Table 2). Although not statistically significant, lower percentages of patients from the dementia groups (dementia only, 6% vs. group with dementia and OP, 8%) went to inpatient rehabilitation.

**Table 2:** Length of stay, hospital charge, and discharge destinations for the four clinical groups

	Clinical Group				
	All	Dementia only	OP only	Both OP and Dementia	Neither OP nor Dementia
	Median (IQR)	Median (IQR)	Median (IQR)	Median (IQR)	Median (IQR)
Length of stay (days)	6 (5–7)	6 (5–7)	6 (5–7)	6 (5–7)	6 (5–8)
Hospital charges ($1000)	45 (3166)	43 (30–68)	46 (32–66)	44 (30–64)	46 (32–66)
Hospital discharge status*	N (%)	N (%)	N (%)	N (%)	N (%)
Home	67 (3%)	12 (4%)	20 (2%)	11 (4%)	24 (2%)
Inpatient Rehab	228 (10.5%)	18 (6%)	91 (11%)	20 (8%)	99 (11%)
SNF/NH	1772 (81.5)	229 (82%)	631 (81%)	201 (81%)	711 (81%)
Acute care	73 (3%)	12 (4%)	26 (3%)	< 10 (1%)	31 (3%)
Died/Hospice	35 (2%)	< 10 (2%)	< 10 (0%)	11 (4%)	10 (1%)

*Abbreviations*: *IQR* interquartile range, *NH* nursing home, *OP* osteoporosis, *SNF* specialized nursing facility

^*^Chi-square test df = 3 (row mean scores differ), statistic = 6.8210, *P* = 0.0778

Table 3 reports the results from the multiple regression analysis showing association of LOS with the four clinical groups. No statistically significant difference was observed among the groups (Kruskal-Wallis, H = 2.79; df = 3; *P* = 0.4243). Having a diagnosis of dementia was associated with a 5% shorter LOS. Similarly, White patients had a 7% shorter LOS, and those with CHF (+ 7%) and IHD (+ 4%) had longer LOS. Unsurprisingly, surgical patients were hospitalized 63% longer than non-surgical patients.

**Table 3:** Results of the multiple regression analysis for hospital length of stay

Parameter	Exponentiated Coefficient (95% Confidence Interval)	***P***
Intercept	3.99 (3.25, 4.91)	<.01
Dementia only	.95 (.90, 1.0)	.04
OP only	1.00 (.97, 1.04)	.85
Both OP and Dementia	.96 (.91, 1.0)	.12
Neither OP nor Dementia	reference	
Female	.98 (.94, 1.02)	.34
Race, White	.93 (.88, .99)	.02
Age at fracture	1.00 (1.00, 1.00)	.24
Congestive heart failure	1.07 (1.04, 1.11)	<.01
Depression	1.00 (.97, 1.03)	.89
Hypertension	1.02 (.96, 1.09)	.56
Hyperlipidemia	.97 (.93, 1.01)	.20
Ischemic heart disease	1.04 (1.00, 1.08)	.03
Arthritis^a^	1.01 (.97, 1.05)	.63
Surgical repair of fracture	1.63 (1.53, 1.74)	<.01

*Abbreviations*: *OP* osteoporosis

^a^Rheumatoid arthritis or osteoarthritis

Table 4 reports results the from the multiple regression analysis showing associations between total hospital charges with the four clinical groups. No statistically significant difference was observed among the four groups (Kruskal-Wallis H = 1.9468; df = 3; *P* = 0.5835). Given that many patient characteristics were associated with group inclusion, we were curious to see how hospital total charges compared across the four groups when adjusting for covariates. All the covariates significantly associated with patient group were included in a regression model. The full model suggests that there is no difference in hospital charges for the four groups after adjusting for sex, race, surgery, and comorbidities. However, after adjusting for group, race, surgery, and comorbidities, hospital charges were 6% lower for women compared to men, and 16% lower for Whites vs. non-Whites. Factors associated with higher hospital charges, after adjusting for group, race, surgery, and comorbidities, were CHF (7% increase), IHD (7% increase), and surgical repair (320% increase).

**Table 4:** Results of the multiple regression analysis for total hospital charges

	Exponentiated Estimate (95% Confidence Interval)	***P***-value
Intercept	23,327.8 (16,831.4, 32,331.6)	<.01
Dementia only	.95 (.87, 1.03)	.18
OP only	1.00 (.95, 1.07)	.90
Both OP and Dementia	1.00 (.92, 1.10)	.91
Neither OP nor Dementia	reference	
Female	.95 (.89, 1.01)	.08
Race, White	.84 (.77, .92)	<.01
Age at fracture	1.00 (.99, 1.00)	.01
Congestive heart failure	1.08 (1.02, 1.14)	.01
Depression	.98 (.93, 1.04)	.54
Hypertension	1.00 (.90, 1.11)	.96
Hyperlipidemia	.99 (.92, 1.05)	.67
Ischemic heart disease	1.07 (1.01, 1.14)	.03
Arthritis^a^	1.04 (.98, 1.10)	.23
Surgical repair of fracture	3.19 (2.88, 3.54)	<.01

*Abbreviations*: *OP* osteoporosis

^a^Rheumatoid arthritis or osteoarthritis

Although there was no statistically significant difference in discharge destination between the groups, most patients (4/5) went to a SNF or NH after discharge. Fewer percentages from the groups with dementia went to inpatient rehabilitation (Table 2).

## Discussion

We report LOS, hospital charges, discharge destination, and chronic comorbidities in Medicare beneficiaries of the USA in four clinical groups based on their diagnosis of OP and dementia prior to hip fracture. Compared to the reference group, patients with dementia only (without OP) had a 5% shorter LOS (*P* = 0.04) when hospitalized for hip fracture. No significant differences were seen in hospital charges between the groups. Patients with CHF and IHD had longer LOS and higher hospital charges than those without CHF and IHD, regardless of their diagnosis of dementia or OP, and being a woman and White race were associated with lower hospital charges. There was a tendency for dementia patients to undergo surgery and be discharged to rehabilitation less frequently than the other groups; however, the trend in discharge status was not statistically significant.

Our findings are contrary to most previous reports about LOS in dementia patients. Studies have reported a longer LOS for patients with dementia in the past [12, 13, 21], which is usually attributed to associated health complications leading to worse health status, including impaired orientation, functional abilities, and risk of fall [13, 15]. Unlike our study, past studies have used data from patients with multiple comorbidities admitted to hospital for acute illnesses not related to hip fracture or OP. Chronic comorbidities were identified to be most likely contributor to increased LOS in patients with dementia worldwide [13]. Patients with CHF and IHD had higher LOS in our study than those without CHF and IHD. Although not statistically significant, prevalence of CHF, IHD, and depression in our study were slightly higher in the dementia group who also suffered from OP. One potential cause of the greater LOS is surgical intervention. The total percentage of patients undergoing surgical intervention in our study was similar to what was reported in Medicare beneficiaries with hip fracture [5]. However, the percentage of patients in the surgical intervention in our study was slightly lower in dementia patients regardless of their OP status. Associations of chronic comorbidities with longer LOS may indicate that LOS is not just an economic parameter but linked to management of medically complex patients.

Coordination of discharge to a facility often takes a longer LOS [22]. There was not much of a difference in percentages of patients between the four clinical groups who were discharged to nursing facilities, including SNFs and NHs. Discharge to home in our study, although low, was higher in dementia related groups (4% in those with dementia alone or with OP vs. 2% in those with OP alone or the reference group). Compared to the dementia-related groups (6–8%), larger percentages of patients (11%) in the OP alone or reference group were discharged to inpatient rehabilitation. These small differences may not be enough to justify a shorter LOS in the patients with dementia in our study. More patients in the OP-related group proceeded to inpatient rehabilitation. No patient in the OP alone group was included in the hospice/mortality group, whereas 2–4% of the dementia-related groups were under this category. A similar percentage of patients in the dementia-related group in the discharge to home or hospice/mortality categories may indicate the frailer physical status of the dementia group. Higher prevalence of frailty is known to be more common among dementia patients with hip fractures [23]. Even without matching percentages, a previous study reported a similar pattern of distribution to discharge destinations for older patients hospitalized for hip fracture with or without some type of dementia [24]. Recent research showed functional improvement in patients with dementia who were sent home with outpatient rehabilitation therapy after the hip fracture event, more so when they had comprehensive geriatric care during rehabiliation [25]. We are limited in knowledge about the rehabilitation status among homebound patients. However, a higher percentage of patients being discharged to SNFs indicates that most of our patients were, physically and cognitively, qualified to participate in physical activity for rehabilitation. Although patients with dementia had a significantly shorter LOS in our study, the average LOS we report is similar to that determined in another study reporting LOS for hip fracture regardless of dementia status [8, 24]. While we are limited in our reporting about patients’ physical and functional abilities, a future study is recommended to explore this aspect of dementia patients with hip fracture, which may affect their health outcomes. One new information our results adds to the literature is that a prior diagnosis of OP did not make a difference in LOS which often linked to patients’ management plan indicating complexity of medical conditions [13].

We did not report significant differences in hospital charges between the four clinical groups. However, women and Whites were more likely to have lower hospital charges that men and non-Whites. One study attributed higher hospital charges for inpatients with dementia due to their comorbidities complicating acute illness and a need for additional in-hospital procedures compared to the hospitalized non-dementia counterparts [12]. Having CHF or IHD posed risk to those having higher charges in our study. As mentioned in previous studies [13], a longer LOS for these patients with CHF and IHD may have contributed to higher hospital charges.

The distribution pattern in types of hip fracture in our study was similar to what was reported previously in older adults, where femoral neck fracture was the most common type (51%), followed by inter-trochanteric fracture (44%) [8]. There were significant differences in fracture types between the four clinical groups. Higher percentages of patients in OP-related groups (OP alone, 48%; OP with dementia, 51%) suffered from inter-trochanteric fractures than in the dementia alone (39%) or reference group (40%). The opposite was noted for femoral neck fractures in our population, with lower percentages in the OP-related groups (OP alone, 46%; OP with dementia, 45%) than in those with dementia alone (57%) and in the reference group (54%). These distributions are consistent with previous study reports that attributed higher incidence of trochanteric fracture in osteoporotic patients due to severe bone loss in this hip region [6]. The mean age of hip fracture in our study is consistent with previous studies that did not clearly report distribution by dementia status [6, 8]. Kim et al. reported the highest incidence of hip fracture in the mid-80s, which ranged between 84 and 88 years of age [6]. Our population showed similar age distribution without any significant differences between the four clinical groups. Although not sharing the source of study patients, our population seems to be similar to those of previous studies in terms of the pattern of osteoporotic fractures and age at the time of the fracture event. However, we add new information as we report these patterns in osteoporotic patients with or without dementia.

### Limitations

There are several limitations with our study. Caution need to be applied when interpreting our results as we report data from the United States which may be different than what is reported in similar developed countries in other continents. However, hip fracture in older adults is a universal health problem; and thus our results may be informative when described with a focus on patients with dementia and other chronic diseases. While we were limited to patients who had an MDS assessment post hip-fracture, we were unable to determine if the admission to the long-term care facilities was related directly to the hip fracture: while over 50% of MDS assessments were performed within a week of discharge, many people were first assessed months later. We do not know if this is due to late reporting or delayed admission. Furthermore, the MDS data were sparsely populated, and we were not able to analyze the MDS data for all the patients who had a hip fracture. We thus cannot rule out other baseline characteristics that may have been associated with outcomes. In addition, we did not determine if the person was living in a long-term care facility at the time of the fracture. Future studies need to consider the facility where the older patients are from to fully understand the extent of the setting’s role in treatment decision making. However, use of Medicare data is a strength for our study. Medicare files are one of the robust claims dataset for older patients in the USA and a source for many health services research as they rely on real world evidence to help improve health care delivery system. One common limitation of all types of administrative/claims data is that they rely on coding and thus the diagnosis is ascertained from those coding in the dataset.

## Conclusion

Our study reports valuable information about hospital outcomes among older Medicare beneficiaries in the USA with dementia and/or OP with hip fractures. To our knowledge, this is the first study reporting LOS in older patients with dementia who suffered a hip fracture. Unlike previous studies on hospital outcomes in dementia patients, we report a 5% shorter LOS in these patients compared to those without any known diagnosis of dementia or OP. A lack of specific information on frailty, level of dementia, and life expectancy post-hip fracture in this particular group limits us from concluding on the reason for a shorter LOS in the dementia group. Regardless of the geographic source of data from the USA, our study brings a new perspective of hospital LOS since previous studies did not look at LOS in dementia in the context of hip fracture. Common comorbidities known for causing a higher LOS (in previous studies) have shown same pattern of LOS as in our study, regardless of clinical grouping. We recommend future studies to confirm our findings and compare similar data from other developed countries.

## Supplementary Information

**Additional file 1: Supplementary Table 1**. ICD-9-CM codes for fracture type and surgical repair.

**Additional file 2: Supplementary Table 2**. Categorization of variables.

## Data Availability

The data that support the findings of this study are available from the Center for Medicare and Medicaid Services but restrictions apply to the availability of these data, which were used under license for the current study, and so are not publicly available. Data are however available from the authors upon reasonable request and with permission of the Center for Medicare and Medicaid Services.

## Abbreviations

LOS: Length of Stay
OP: Osteoporosis
CMS: Centers for Medicare and Medicaid Services
MDS: Minimum dataset
SNF: Skilled Nursing Facility
NH: Nursing homes
CHF: Congestive heart failure
IHD: Ischemic heart disease
AMI: Acute myocardial infarction
CKD: Chronic kidney disease
CCI: Charlson Comorbidity Index
