# Supplementary material for: Shorter length of hospital stay for hip fracture in those with dementia and without a known diagnosis of osteoporosis in the USA
Source: BMC Geriatr. 2020 Dec 3;20:523. doi: 10.1186/s12877-020-01924-x (PMC7713172; doi:10.1186/s12877-020-01924-x)
Supplement: Supplementary file 1 — Additional file 1: Supplementary Table 1. ICD-9-CM codes for fracture type and surgical repair. [file 12877_2020_1924_MOESM1_ESM.docx]

**Supplementary Table 1. ICD-9-CM codes for fracture type and surgical repair**

| Fracture type | ICD-9-CM diagnosis or procedure codes |
| --- | --- |
| femoral neck | 820.00, 820.01, 820.01, 820.03, 820.09, 820.10, 820.11, 820.12, 820.13, 820.19, 820.20, 820.30, 820.8, 820.9 |
| intertrochanteric | 820.21, 820.31 |
| subtrochanteric | 820.22, 820.32 |
| Surgical repair | 00.74, 00.75, 00.76, 00.77, 00.85, 00.86, 00.87, 78.55, 79.15, 79.25, 79.35, 79.55, 79.65, 79.85, 79.95, 81.52, 81.40, 81.51 |
