# Supplementary material for: Shorter length of hospital stay for hip fracture in those with dementia and without a known diagnosis of osteoporosis in the USA
Source: BMC Geriatr. 2020 Dec 3;20:523. doi: 10.1186/s12877-020-01924-x (PMC7713172; doi:10.1186/s12877-020-01924-x)
Supplement: Supplementary file 2 — Additional file 2: Supplementary Table 2. Categorization of variables. [file 12877_2020_1924_MOESM2_ESM.docx]

**Supplementary Table 2. Categorization of variables**

| **Category created** | **Codes used** |
| --- | --- |
| Race, category assigned | Bene_race_cd |
| White | 1 |
| Other | 0, 2, 3, 4, 5, 6 |
| Discharge status category assigned | ptnt_dschrg_stus_cd |
| Home | 01, 04, 06, 07, 08, 21, 65, 71, 72, 86, 87, 93 |
| Inpatient Rehab | 62, 90, 91 |
| SNF/NH | 03, 61, 64, 83, 84, 89, 90, 91, 92, |
| Acute care | 02, 05, 09, 30, 43, 63, 66, 69, 70, 82, 85, 88, 94, 95 |
| Died/Hospice | 20, 40, 41, 42, 50, 51 |

Abbreviations: NH, nursing home; SNF, specialized nursing facility.
